# Protective Effect of *Aloe vera* Extract against Bisphenol A Induced Testicular Toxicity in Wistar Rats 

**DOI:** 10.22074/cellj.2018.5256

**Published:** 2018-03-18

**Authors:** Mohammad Amin Behmanesh, Hosein Najafzadehvarzi, Seyedeh Mahsa Poormoosavi

**Affiliations:** 1 Department of Histology, School of Medicine, Dezful University of Medical Sciences, Dezful, Iran; 2 Department of Pharmacology, Faculty of Medicine, Babol University of Medical Sciences, Babol, Iran

**Keywords:** *Aloe vera*, Bisphenol A, Rat, Testis

## Abstract

**Objective:**

Bisphenol A (BPA), an endocrine-disrupting chemical, has been considered as a possible risk factor for fertility because it induces testicular toxicity. Thus, we sought to analyze the effect of *Aloe vera* as plant with antioxidant properties on tissues and oxidative stress parameters in male rats.

**Materials and Methods:**

In this experimental study, 50 adult male Wistar rats (200 ± 20 g) have been used in this 56 day study. Animals were completely randomized and divided into five groups: A_1_(control), A_2_(vehicle control), A3 (*Aloe vera* gel 300 mg/kg), B_1_(BPA 20 µg/kg bw) and B_2_(*Aloe vera* gel+ BPA). At the end of the study, the rats were anesthetized and 2 ml blood samples were obtained for evaluation of oxidative stress markers. Also, both testes were collected for histological examinations.

**Results:**

BPA significantly decreased (P<0.05) body and testis weights. Seminiferous tubule diameter (STD) and height of seminiferous epithelium (HSE), were significantly decreased (P<0.05) in the groups receiving BPA as compared to the control. There was also a reduction in the quantity of spermatocyte and spermatids. Moreover, malondialdehyde (MDA) increased and thiol protein (G-SH) decreased. But, co-administration of *Aloe vera* with BPA accelerated the total antioxidant capacity and testicular tissue structure healing.

**Conclusion:**

According to our findings, *Aloe vera* gel extract can overcome the damaging effects of BPA on the reproductive system of rats and protects rats’ testes against BPA-induced toxicity.

## Introduction

Bisphenol A (BPA) is a potential endocrine-disrupting chemical because it has the capability to imitate the activity of natural estrogen. It is used in metal cans produce for food packaging or for coating metal lids of glass jars and bottles. It is also found in some plastic food boxes, all disposable plastic stuffs, toys, dental gadgets and sealants (1). It can leak into food, air or water. Beside its endocrine disrupting effect, it is also known that BPA induces oxidative stress and causes toxicity in the reproductive system (2). 

Consequently, attention has been drawn towards the effects of low doses of BPA on human development and reproduction (3) and its toxicity in the reproductive system components such as testis and epididymis as well as sperm count, in male rats. Many herbs and plant products have been shown to have antioxidant action, which have led to increasing demand for herbal products with antioxidant and disease preventive properties which posse lower side effects.

*Aloe vera* is one of these antioxidant plants (4) which has stiff gray-green lance-shaped leaves containing clear gel in a central mucilaginous pulp (5). In some studies, it has been shown that *Aloe vera* has an antioxidative effect, can improve spermatogenesis and has positive effect on testosterone and histological features of the testis (6).

So, the present experimental study was designed with a histological approach to demonstrate the impact of BPA on testis injury and possible protective effect of *Aloe vera* against BPA-induced alteration in Wistar rat’s testis. Also, to determine serum concentration of oxidative stress indices such as malondialdehyde (MDA) and thiol protein (G-SH), were determined following BPA administration and its co-administration with *Aloe vera*. 

## Materials and Methods

### Animal housing

In this experimental study, 50 adult male Wistar rats (10-14 weeks old, 200 ± 20 g) were obtained from Dezful University of Medical Sciences Animal house (Dezful, Iran) and were used in a completely randomized design. During the study, all rats were kept under controlled light and dark condition (12-hour light/12-hour dark cycle) at room temperature (25 ± 2°C), and had free access to food and water. The study was approved by the Ethics Committee of Dezful University, and all the experiments were performed in accordance with the guidelines for the safe handling of animals.

### Animal grouping 

Animals were randomly divided into two groups: group A which served as the control group and included thirty adult male rats that were subdivided into: subgroup A_1_ included ten adult male rats which received a daily oral gavage of normal saline, subgroup A_2_ included ten adult male rats which received a daily oral gavage of olive oil (5 ml/kg) as vehicle (7), subgroup A_3_ included ten adult male rats which received a daily oral gavage of *Aloe vera* gel (400 mg/kg) (8) and group B which included twenty adult male rats that were subdivided into two equal subgroups (10 rats each): subgroup B1 received oral BPA dissolved in 5 ml/kg olive oil daily at a dose of 20 µg/ kg (2), subgroup B_2_ received similar doses of BPA orally along with 400 mg/kg *Aloe vera* gel. Bisphenol A (CAS No. 80-05-7 with a purity of 97%) was purchased from Sigma-Aldrich Company (USA) and dissolved in olive oil (as the vehicle). The *Aloe vera* gel was prepared by Barij Esance Company (Iran). All treatments were given daily by an oral gavage, for 8 weeks (1).

### Samples collection

At the end of the experiment, the rats were weighed, anaesthetized with sodium thiopental (30 mg/kg) and sacrificed. Then, the testis was removed and weighed. Blood samples were collected from the heart using a 2-ml syringe without using anticoagulant agents and centrifuged at 3000 rpm for 10 minutes for serum separation.

### Serum biochemical measurement

The collected serum samples were utilized for the measurement of biochemical markers such as lipid peroxidation marker, MDA that reacts with thiobarbituric acid in a test tube, produces a red complex, and is measured by spectrophotometry. The intensity of the color is proportional to MDA levels in serum and ultimately to the oxidative stress (9). Also, in order to measure G-SH, the Elman indicator (5, 5’-dithiobis-(2-nitrobenzoic acid) (DTNB) was used. DTNS reacts with reduced sulfhydryl groups (G-SH) and forms a colored complex, which can be measured by spectrophotometry (5) at 450 nm. All of the above-mentioned chemicals were purchased from Merck, Germany.

### Preparing testis tissue

The testis samples were fixed in 10% formalin. The 5-6 µm sections were prepared using paraffin embedding techniques by rotary microtome (RM2235, Leica Company from USA) and stained with hematoxylin-eosin for the histological examination.

### Histomorphometry 

In the histomorphometric study, photos were taken from sections using an Olympus optical microscope equipped with a Dino lit camera at the magnifications of ×4, ×10 and ×40 investigating four random points. Dino lit software was used for extracting the data. The diameter of seminiferous tubules at a magnification of ×10, were measured in each animal and 5 sections from one animal in each group, the 3 seminiferous tubules were measured from 5 randomly chosen areas, and for each tubule, long diameters were measured by Dino lit software. Diameter and height of the epithelium of seminiferous tubules were also measured in each animal.

### Detemination the number of primary spermatocytes, spermatogonia and spermatids

In order to count the number of primary spermatocytes, spermatogonia and spermatids in 1 cm (2), the figures at a magnification of ×40 were used as above mentioned. All of the above protocols were done using Dino Lit software.

### Statistical analysis 

All the analyses were performed using SPSS version 16. Group’s variance were analyzed by one-way Analysis of Variation (ANOVA) and Fisher’s least significant difference test (LSD) for evaluation of significant differences between groups. A P<0.05 was considered statistically significant. 

## Results

The statistical analysis showed a significant decrease (P<0.05) in the weight of the rats after BPA consumption compared to the control group. Furthermore, no significant (P<0.05) difference was seen between the other groups and control group. The mean weights of the rats in the control, Olive oil, BPA, *Aloe vera* and BPA+*Aloe vera* groups on the last day of the experiment are shown in Table 1. 

**Table 1:** Mean ± SD of the testis weight and body weight of rats in different groups


Parameters	Weight of testis (g)	Body weight (g)
Group		

Group A_1_	1.5 ± 0.5^a^	240 ± 1.5^a^
Group A_2_	1.4 ± 1.42^a^	234 ± 1.5^a^
Group A_3_	1.3 ± 1.31^a^	230.3 ± 1.4^a^
Group B_1_	1.1 ± 0.6^b^	181.1 ± 3.1^b^
Group B_2_	1.29 ± 2.1^a^	228 ± 2.7^a^


^a, b^; In each column indicate significant differences at P<0.05.

Gross examination of the testes did not show any deformities in any animals of any group. The testicular weight values were significantly (P<0.05) lower in the BPA-administered rats, compared to the control group. However, the testicular weight values were significantly (P<0.05) higher in the BPA+*Aloe vera* group compared to the BPA group (Table 1). BPA administration produced significant (P<0.05) changes in the oxidative stress parameters in the serum of rats as shown by increased MDA and decreased G-SH content (Table 2). 

**Table 2:** Mean ± SD of the malondialdehyde (MDA) and thiol protein (GSH) levels in rats


Parameters	MDA (nmol/ml)	GSH (µmol/ml)
Group		

Group A_1_	420.9 ± 3.2^c^	31.2 ± 0.9^a^
Group A_2_	413 ± 2.9^c^	32.7 ± 2.6^a^
Group A_3_	425.6 ± 6.1^c^	33.3 ± 6.1^a^
Group B_1_	775.6 ± 6.1^a^	21. 6 ± 5.2^b^
Group B_2_	536.1 ± 3.4^a^^b^	29.2 ± 3.4^a^


^a, b^ and ^c^ in each column indicate significant differences at P<0.05.

Morphometric findings of testis are shown as seminiferous tubule diameter (STD) (µm), height of seminiferous epithelium (HSE), as well as the number of spermatogonia, primary spermatocytes and spermatids (Tables3, 4). 

**Table 3:** Mean ± SD of the diameter of seminiferous tubule and thickness of epithelium in different groups


Parameters	Diameter (µm)	Thickness (µm)
Group		

Group A_1_	231 ± 1.5^a^	71 ± 1.2^a^
Group A_2_	234 ± 1.4^a^	69.2 ± 1.6^a^
Group A_3_	230.3 ± 1.4^a^	68.2 ± 2.3^a^
Group B_1_	161 ± 3.1^b^	39.2 ± 1.8^c^
Group B_2_	198 ± 2.1^a^	56.2 ± 1.3^b^


^a, b^ and ^c^ in each column indicate significant differences at P<0.05.

**Table 4:** Mean ± SD of the number of spermatids, primary spermatocyte and spermatogonia in rats


Parameters	Spermatid	Primary spermatocyte	Spermatogonia
Group			

Group A_1_	16.2 ± 4.1^a^	12.4 ± 2.2^a^	11.1 ± 2.1^a^
Group A_2_	16.5 ± 2.5^a^	12.6 ± 2.6^a^	10.2 ± 1.3^a^
Group A_3_	13.4 ± 2.7^a^	12.6 ± 3.2^a^	10.8 ± 3.3^a^
Group B_1_	7.5 ± 2.1^b^	9.93 ± 3.7^b^	10.6 ± 3.1^a^
Group B_2_	14.2 ± 3.3^a^	11.9 ± 3.4^a^	11 ± 1.3^a^


^a, b^ and ^c^ in each column indicate significant differences at P<0.05.

**Fig.1:**
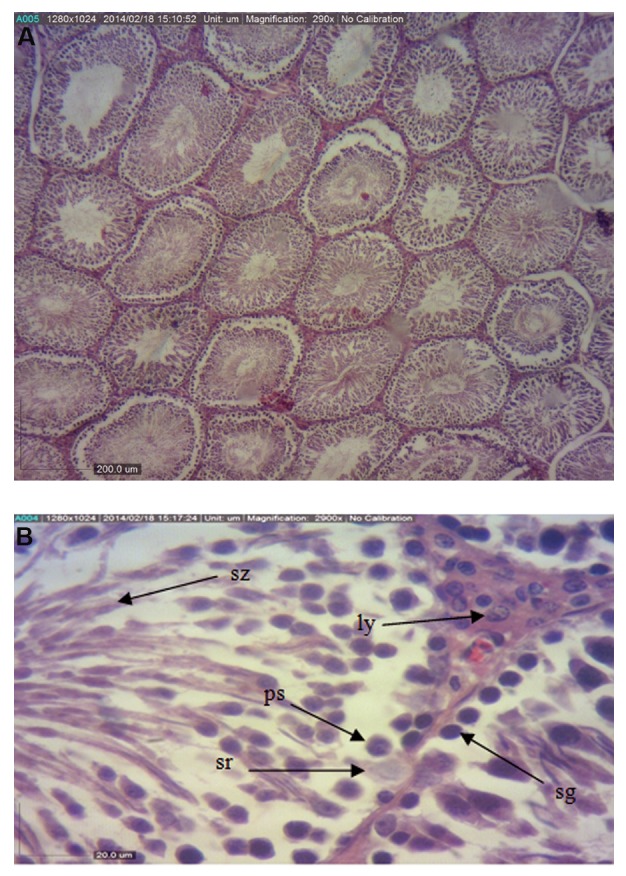
Light photomicrographs of the testis in control group. A. The seminiferous tubules have normal shape and ordinary height of epithelium (H&E, ×40) and B. (H&E, ×400). Sz; Spermatozoa, ps; Primary spermatocyte, sg; Spermatogonia, and sr; Sertoli.

**Fig.2:**
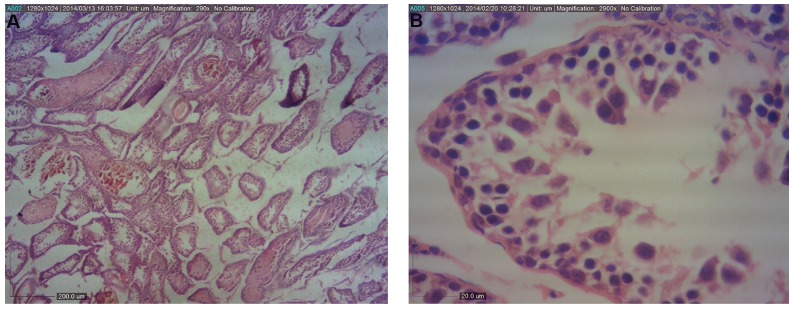
Light photomicrographs of the testis in BPA-received group. A. The seminiferous tubules have irregular shape and epithelium is desultory (H&E, ×40) and B. (H&E, ×400).

**Fig.3:**
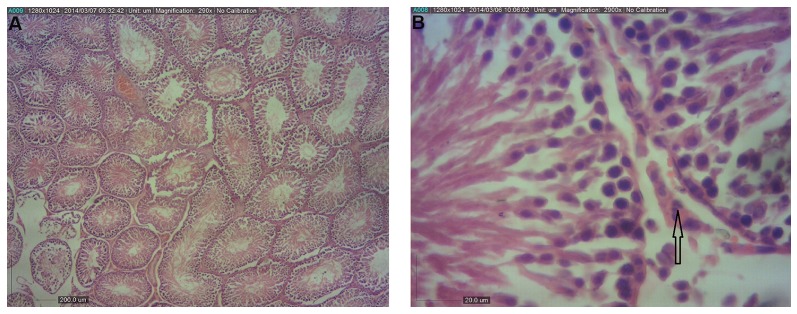
Light photomicrographs of the testis in group that received BPA and *Aloe vera*. A. The seminiferous tubules have normal shape and ordinary height of epithelium, showing minimal amount of intertubular connective tissues (arrow) (H&E, ×40), B. (H&E, ×400).

The values of the diameter of seminiferous tubules in vehicle, control (Fig .1), and *Aloe vera* groups were significantly higher (P<0.05) compared to the BPA group (Fig .2). Also, HSE was increased in BPA+*Aloe vera* group compared to the BPA group (Fig .3). The height of the epithelium was significantly (P<0.05) higher in the control group compared to the BPA while the height of the epithelium in other groups was not significantly different (P<0.05) from the control group. 

The number of spermatocytes, spermatogonia and spermatids in the seminiferous tubules was counted; the results showed no significant (P<0.05) difference between the average number of spermatogonial cells in different groups, but the primary spermatocytes and spermatids in different groups significantly varried as the minimum number of primary spermatocytes and spermatids cells was observed in BPA group that was significantly (P<0.05) different comparing to the other group, but the amount of spermatogonial cells had no significant (P<0.05) difference between the groups (Table 4). 

Treatment with *Aloe vera* ameliorated the histological damage induce by BPA in the testes’ as histological evaluations showed signs of revival and the number of primary spermatocytes and spermatids greatly increased (Fig .3). 

## Discussion

In recent years, endocrine-disrupting chemicals, such as BPA which is released into the environment, has attracted considerable attention (10). Exposure to BPA causes oxidative damage in tissues, but this damage can be diminished by using antioxidants (11).

*Aloe vera* has antioxidant properties that might be beneficial for decreasing the toxic impact of BPA. However, there is no investigation concerning the effect of *Aloe vera* on endocrine disrupting chemicals-induced oxidative stress in the tissues (12). Doses of *Aloe vera* that were used in this study are very high as compared to other studies and were selected according to a recent research done by Behmanesh et al. (8). Also, in this experiment, BPA was used at the dose of 20 µg/kg to affect the reproductive system (2).

Based on our data, BPA decreased the body weight of animals. Nanjappa et al. (11) reported that the final body weights of rats significantly decrease following exposure to BPA. Moreover, it was reported that a high dose of BPA (400 mg/kg) significantly decreases the body weight of rats. However, few research indicated that the body weight of male rats given 0.2, 2 and 20 µg/kg BPA did not show significant changes (13).

In the present study, weight gain was observed in control group but, considerable weight loss in BPA-treated groups may confirm that BPA affects the metabolic activity of the animals following long-term exposure (14). Also, regarding the body weight loss due to administration of BPA, Isidori et al. (15) found that toxic stresses can cause a decrease in the testosterone level by lowering the antioxidant capacity and blocking P450 cytochrome 17. In this regard, the decrease in the level of testosterone could induce weight loss by decreasing muscles and bone mass. Our results revealed that BPA-induced oxidative and toxic damages, can be decreased by antioxidants. *Aloe vera* has antioxidant properties that may decrease the toxic effects of BPA. Other studies have also investigated this hypothesis by using similar plants with antioxidant activities (16, 17).

The results of the present study showed that the diameter of seminiferous tubule and thickness of epithelium reduced following exposure to BPA compared to other group because of low serum testosterone level and BPA toxicity (18), and due to reduction of significant decrease in the paired weight of testes. Reductions in the weight of the reproductive organs were also due to small numbers of primary spermatocytes and spermatids because spermatogonia need testosterone to begin their function, and BPA-treated animals had very low levels of this hormone (19), which indicate the cause of spermatogenesis failure. 

Results of the present study are consistent to those reported by Norazit et al. (13) and Chitra et al. (2). Generally, some studies showed non-significant differences in testes or body weight of rats (20) while other indicated significant changes in body weight and relative organs weight (21). These discrepancies in weight changes may be related to the differences in doses, routes of administration, duration, and time of exposure, rat strains, sex and nature of food (19).

All histopathological changes that discussed in the above-mentioned sentences, were ameliorated when *Aloe vera* was co-administered with BPA, because according to some studies, flavonoids present in *Aloe vera* extract can increase the level of testosterone; also, antioxidant compounds found in *Aloe vera* (especially vitamin E) prohibit reductions in the number of Leydig and Sertoli cells. Also, vitamin E improves testis weight, seminiferous tubule diameter and thickness of the germinal epithelium (22). Furthermore, data from present study likely revealed that BPA exposure disrupts oxidant-antioxidant balance in male rats so that the levels of MDA significantly increased but levels of G-SH significantly decreased.

The present findings confirmed the results of a previous study conducted by Moghaddam et al. (23). *Aloe vera* was found to be effective in increasing the G-SH and decreasing the MDA compared to the BPA-fed group confirming the previously recorded antioxidant effect of *Aloe vera* (8), due to its phenolic and flavonoids contents (24). Also, Haritha et al. (25) showed that *Aloe vera* can reduce highly reactive oxygen species that can cause extensive damage to cell membranes lipids, and decrease MDA, which is in consitency with our study.

The higher levels of G-SH in rats which received Aloe vera, show the plant’s potentials in reducing the damaging effects of toxic substances, in male reproduction system, suggesting its possible application for improvement of fertility. 

## Conclusion

The results of this study demonstrated that BPA can destruct the testis tissue and reduce spermatogenesis and provided further evidence on the oxidative adverse effect of BPA in testis that can lead to infertility. Treatment with *Aloe vera* gel showed a protective effect against such adverse effects. Therefore, it is recommended to supplement *Aloe vera* gel for male individuals suffering from infertility.
